# High-Fidelity Prototyping for Mobile Electronic Data Collection Forms Through Design and User Evaluation

**DOI:** 10.2196/11852

**Published:** 2019-03-22

**Authors:** Alice Mugisha, Ankica Babic, Peter Wakholi, Thorkild Tylleskär

**Affiliations:** 1 Center for International Health Department of Global Public Health and Primary Care University of Bergen Bergen Norway; 2 Department of Information Technology School of Computing and Informatics Technology Makerere University Kampala Uganda; 3 Department of Information and Media Studies Faculty of Social Sciences University of Bergen Bergen Norway; 4 Department of Biomedical Engineering University of Linköping Linköping Sweden

**Keywords:** high-fidelity prototype, group user testing, mobile electronic data collection forms, usability evaluation

## Abstract

**Background:**

Mobile data collection systems are often difficult to use for nontechnical or novice users. This can be attributed to the fact that developers of such tools do not adequately involve end users in the design and development of product features and functions, which often creates interaction challenges.

**Objective:**

The main objective of this study was to assess the guidelines for form design using high-fidelity prototypes developed based on end-user preferences. We also sought to investigate the association between the results from the System Usability Scale (SUS) and those from the Study Tailored Evaluation Questionnaire (STEQ) after the evaluation. In addition, we sought to recommend some practical guidelines for the implementation of the group testing approach particularly in low-resource settings during mobile form design.

**Methods:**

We developed a Web-based high-fidelity prototype using Axure RP 8. A total of 30 research assistants (RAs) evaluated this prototype in March 2018 by completing the given tasks during 1 common session. An STEQ comprising 13 affirmative statements and the commonly used and validated SUS were administered to evaluate the usability and user experience after interaction with the prototype. The STEQ evaluation was summarized using frequencies in an Excel sheet while the SUS scores were calculated based on whether the statement was positive (user selection minus 1) or negative (5 minus user selection). These were summed up and the score contributions multiplied by 2.5 to give the overall form usability from each participant.

**Results:**

Of the RAs, 80% (24/30) appreciated the form progress indication, found the form navigation easy, and were satisfied with the error messages. The results gave a SUS average score of 70.4 (SD 11.7), which is above the recommended average SUS score of 68, meaning that the usability of the prototype was above average. The scores from the STEQ, on the other hand, indicated a 70% (21/30) level of agreement with the affirmative evaluation statements. The results from the 2 instruments indicated a fair level of user satisfaction and a strong positive association as shown by the Pearson correlation value of .623 (*P*<.01).

**Conclusions:**

A high-fidelity prototype was used to give the users experience with a product they would likely use in their work. Group testing was done because of scarcity of resources such as costs and time involved especially in low-income countries. If embraced, this approach could help assess user needs of the diverse user groups. With proper preparation and the right infrastructure at an affordable cost, usability testing could lead to the development of highly usable forms. The study thus makes recommendations on the practical guidelines for the implementation of the group testing approach particularly in low-resource settings during mobile form design.

## Introduction

### Background

Usability implementation in many design scenarios, even in user-centered designs (UCDs), is still unsatisfactory [1]. This leads to unusable interfaces especially for nontechnical users [2], and such interfaces contribute to the failure of most interactive systems [3]. Of the reasons for this failure, 1 is that developers of open-source software (OSS) such as the mobile electronic data collection forms (MEDCFs) are not prioritizing the use of the UCD approach in their software development projects. They instead develop software targeting particular features [4]. This approach often leaves out the end users in the design and evaluation of these systems, whose major role is to interact with the finished products. As a result, in low- and middle-income regions, several data collection systems exist, but these are often difficult to deploy, hard to use, complicated to scale, and rarely customizable [5], hence grossly decreasing their usability.

The mobile user interface designs are usually based on the desktop paradigm whose designs do not fully fit the mobile context [6], which in turn breeds usability challenges. Other challenges may also be hardware related, for example mobile phones have limited disk space, memory, processor speed, and battery life, among others. In addition, the mobile networks on which they depend are highly variable in performance and reliability [7]. Furthermore, the limited screen size makes efficient presentation of information and navigation to the users difficult [8,9]. In fact, some of the electronic forms have multiple questions, which may make presentation on the screen quite complicated. In some phones, the display resolution may not favor good presentation of tables and images on the screen. Additionally, the keyboard size or character setting is limited irrespective of the users’ finger size [10,11] and the content. This leads to incorrect choice selection and wastage of time in additional scrolling activities, which is also common with smaller interfaces [10,12].

### Literature Studies and Justification

Usability is mainly concerned with the exhibited design features of interactive products in relation to how easy the user interface is to use [13], as well as the user satisfaction as a result of such use [14]. Usability is, therefore, defined by characteristics such as the cognitive perception, the ability to interact with the system, and the perception of the response from the system [3], which may vary across individuals. Important to note is that the usability of MEDCFs relies on the capabilities of the software provided by the software developers [15]; however, a number of developers have a limited understanding of usability [1,2] and how it can be implemented. This is because despite the fact that the developers’ goal is usability, they tend to follow engineering criteria, which results in products that seem obvious in their functioning for the developers but not for general users, and this often leads to negative results after evaluation [16,17]. Evaluation is one of the primary stages in the UCD and in design science research (DSR), which can be used to improve the quality of any system or prototype during and after its development. Evaluation is essential in conducting rigorous DSR as it provides evidence that a newly created artifact achieves the purpose for which it was designed [18]. However, evaluating usability alone may not be sufficient to improve the quality of the system, without considering the emotions and feelings of the users as they interact with the systems or applications [19]. This brings in the aspect of user experience (UX), which is concerned with getting a more comprehensive understanding of the users’ interactive experiences with products or systems [20]. UX includes all the users’ emotions, preferences, perceptions, behaviors, and accomplishments that occur before (preinteraction experience), during (actual interaction experience), and after use (postinteraction experience) of the product [19-21].

User testing is one of the usability evaluation methods where the assessment of the usability of a system is determined by observing the users working with that system [22]. Here, a representative number of end users perform a set of tasks using a prototype system, and the usability challenges are presumably identified by user observations during the exercise [23]. Group usability testing, on the other hand, also involves several participants individually but simultaneously performing the given tasks, with one or more testers observing and interacting with the participants [24]. The motivation for testing is based on the assumption that any system that is designed for people to use should be easy to learn and remember, contain the functions that people really need in their work, and also be easy and pleasant to use [25]. Evaluating user design preferences is not a common approach in the development of mobile data collection forms partly because of time and financial constraints. In fact, this is the first study in Uganda where this kind of testing has been conducted, and we do not have knowledge of any such study from the published literature.

### Objectives

This study therefore assesses a set of design guidelines using the group testing approach and records the end users’ experience after interacting with the high-fidelity prototype. It also recommends some practical ways of implementing group testing during mobile form design, particularly in low-resource settings. To achieve this, a high-fidelity prototype was developed based on the end users’ design preferences and evaluated by the research assistants (RAs) for usability and UX after interaction using SUS and STEQ. We report the level of satisfaction and the features from the prototype the RAs are satisfied with.

## Methods

### Participants

The study participants were 30 RAs, and all of them were collecting data on a maternal and child health project (the Survival Pluss project) in northern Uganda, which is funded by the Norwegian Programme for Capacity Development in Higher Education and Research for Development (NORHED) [26]. Of the RAs, 3 were certificate holders and 9 were diploma holders, whereas 18 were degree holders in various fields, which included accounting, agriculture, social work, laboratory services, and nursing. Of these, 23 RAs had been collecting data for a period of 2 years or less, whereas 7 had collected data for a period ranging from 4 to 6 years. All the RAs had used open data kit (ODK) [5,27] to collect data; however, 3 reported to have used tangerine, Survey Monkey, and OpenMRS, in addition to ODK [28].

### Prototype

A Web-based high-fidelity prototype for MEDCFs was developed between January and February 2018. This prototype was meant to demonstrate the RAs’ design preferences having collected them earlier using a mid-fidelity prototype [29,30]. It was also used as a basis for evaluating to what extent these design preferences contribute to the usability of the data collection forms. A high-fidelity prototype is a computer-based interactive representation of the product with a close resemblance to the final design in terms of details and functionality. The high-fidelity prototypes not only test the visuals and aesthetics of a product but also the UX aspects in relation to interaction with the product [31]. The prototype (see Multimedia Appendix 1) was created in Axure RP 8 without any backend functionality and was created to fit on Samsung Galaxy J1 Ace phones that were being used to collect data on the Survival Pluss project, and they had a view port size of 320 by 452.

The prototype had 3 main sections structured based on the project’s content. These consisted of the demographic section where participants were required to fill the participant ID, interviewer name, and interviewer telephone number. Section I had list pickers and section II showed different table designs capturing a child’s sickness record. We explained to the RAs the potential value of the user testing exercise before giving them access to the prototype and to the tasks they were supposed to do. A summary of the entered data on the child sickness was available for the users to crosscheck and *agree* or *disagree* to its correctness, after which they were prompted to submit. Before submission, the users were warned of the inability to edit the data once they have been submitted. At this point, the progress bar indicated 100%, meaning that the form had been filled to completion and submitted.

### Group Testing Exercise

The group testing exercise was conducted in February 2018 in Lira, Uganda. The RAs were required to complete some tasks (Multimedia Appendix 2) during the group testing exercise. This was meant to create uniformity in the prototype evaluation and also to be able to measure the time it took for each of the RAs to complete the same tasks. In addition to carrying out the tasks, they were also meant to read the feedback given as a result of the actions carried out and to respond appropriately until they correctly submitted the form. It was a requirement to complete all the tasks before submission of the form, and the participants were expected to record their start time before and finish time after the testing exercise. A total of 2 observers were present to record the exercise and to attend to the questions when asked to. The start time and end time were recorded for each participant in each session.

### Prototype Evaluation

The prototype evaluation happened immediately after the group testing exercise. This was an ex-post naturalistic evaluation because we were evaluating an instantiated artifact in its real environment, that is, with the actual users and in the real setting [18,32]. The artifact was a high-fidelity prototype, and the actual users were the RAs who were collecting data on mobile phones using ODK, an OSS software.

### Instruments Used in the Prototype Evaluation

A total of 2 instruments were used to evaluate the prototype usability, one was the SUS, a standardized questionnaire, and the other was STEQ. By combining the two, we expected to gain more detailed insight and also to test our generated questionnaire against the standardized one. These 2 posttest questionnaires were administered after the participants had completed the tasks in a bid to show how users perceived the usability of the data collection forms [33].

The STEQ comprised 13 statements and was developed based on the literature with a purpose of making an alternative instrument, other than the SUS. The statements were based on features such as form progress, simplicity in use, error correction and recovery, and visual appeal, among others. The RAs were required to indicate their level of agreement with the evaluation statements by selecting options, which included *strongly disagree*, *disagree*, *somewhat agree*, *agree*, *strongly agree*, and *don’t know* and were tallied to a score of 1, 2, 3, 4, 5, and 6, respectively. The evaluation statements were selected from 4 usability evaluation questionnaires, namely the Computer System Usability Questionnaire [34], Form Usability Scale [35], Questionnaire for User Interaction Satisfaction [36], and statements from the Usability Professional Association [37]. The selected statements were based on the fact that they could be used to assess usability in mobile data collection forms as defined by the design preferences of the RAs and were all affirmative statements with positive valence. It is alleged that participants are less likely to make mistakes by agreeing to negative statements [38] similar to the case of a balanced questionnaire consisting of positive and negative statements [39]. However, and for the sake of simplicity, we used only affirmative statements adopting the style of the 4 abovementioned usability evaluation questionnaires.

The SUS is a balanced questionnaire that is used to evaluate the usability of a system and comprises 10 alternating positive and negative statements [40]. The SUS acted as a complementary scale to the STEQ. The SUS has been experimentally proven to be reliable and valid [33] because of its ability to control against acquiescence bias and extreme response bias [38,39]. In acquiescence bias, respondents tend to agree with all or almost all statements in a questionnaire, whereas the extreme response bias is the tendency to mark the extremes of rating scales, rather than the points near the middle of the scale [38,39]. These biases greatly affect the true measure of an attitude. The word *system* was replaced with the word *form* for some of the statements in both questionnaires.

**Table 1:** The 13 statements in the tailormade evaluation questionnaire and the number of respondents (n=30) in each category from *strongly disagree* to *strongly agree*.

Evaluation statement	Strongly disagree, n (%)	Disagree, n (%)	Neutral, n (%)	Agree, n (%)	Somewhat agree, n (%)	Don’t agree, n (%)	Total (N)^a^
The form informs about its progress during interaction	0 (0)	0 (0)	2 (6)	8 (27)	20 (67)	0 (0)	30
The information, for example, onscreen messages provided in this form were clear	1(3)	0 (0)	3 (11)	4 (14)	18 (64)	2 (7)	28
It was easy to move from one page to another	3 (10)	2 (6)	1 (3)	8 (27)	15 (50)	1 (3)	30
The overall organization of the form is easy to understand	1 (3)	0 (0)	2 (6)	13 (43)	12 (40)	1 (3)	30
I knew at every input what rule I had to stick to (possible answer length, date format, etc)	2 (6)	3 (10)	7 (23)	5 (17)	13 (43)	0 (0)	30
Reading of characters on the form screen is easy	1 (0)	3 (10)	9 (30)	17 (57)	0 (0)	0 (0)	30
The form gave error messages that clearly told me how to fix the problems	3 (10)	1 (3)	1 (3)	2 (6)	21 (70)	2 (6)	30
I was able to fill in the form quickly	2 (6)	4 (13)	3 (10)	8 (27)	13 (43)	1 (3)	30
It was simple to fill this form	1 (3)	1 (3)	5 (17)	10 (33)	13 (43)	0 (0)	30
Whenever I made a mistake when filling the form I could recover easily and quickly	0 (0)	1 (3)	2 (6)	5 (17)	21 (70)	1 (3)	30
This form is visually appealing	0 (0)	2 (6)	6 (20)	10 (33)	10 (33)	2 (6)	30
Overall, the form is easy to use	1 (3)	2 (6)	1 (3)	8 (27)	17 (57)	1 (3)	30
Overall, I am satisfied with this form	0 (0)	0 (0)	7 (21)	8 (27)	14 (41)	1 (3)	30

^a^Some respondents did not reply to all statements.

Results from the 2 instruments were compared. Previous studies have shown that irrespective of the questionnaires used being balanced or affirmative, the scores from the 2 questionnaires are likely to be similar [38]. This is because there is little evidence to show that the advantages of using balanced questionnaires outweigh the disadvantages, some of which include misinterpretation of the scales leading to mistakes by the users [38]. The STEQ was summarized using frequencies in an Excel sheet where the evaluation statement with majority *agreeing* to it was taken as the option which RAs were most satisfied with (Table 1). On the other hand, SUS scores are calculated based on the statement being scored [40], and we did the same in this study. For the positive statements 1, 3, 5, 7, and 9, the score contribution was what the user had selected minus 1. For the negative statements 2, 4, 6, 8, and 10, the score contribution was 5 minus what the user had selected. The total sum of the score contributions was obtained and multiplied by 2.5 [40]. This gave the overall result of the form usability from each participant.

## Results

This section presents the results after evaluation of the high-fidelity prototype using the tailor-made evaluation questionnaire and the SUS.

### End-User Experience in Relation to System Usability Scale and Study Tailored Evaluation Questionnaire Scores

Of the data RAs, 80% (24/30) *agreed* that the form progress was visible, form navigation and organization were easy, and that the error messages clearly indicated how to fix problems. The same number also *agreed* that the form was simple, that it was quick and easy to recover in case of a mistake, and that overall the form was easy to use. In addition, half of the participants also *agreed* that they knew the rules to stick to when inputting the data and also found reading characters on the form easy.

**Figure 1:**
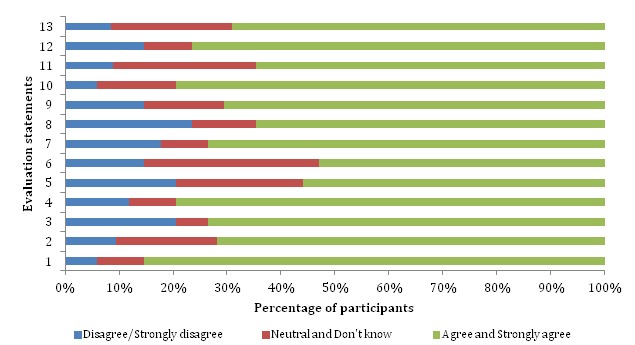
The percentage of participants who agreed, disagreed or were neutral to the evaluation statements.

However, more than 23% (7/30) of the participants *disagreed* to the form being easy to navigate and to the ability to fill the form quickly. Still some of the participants were neutral to some of these evaluation statements, that is, they neither *agreed nor disagreed*. For example, 36% (11/30) of the participants were neutral about easy reading of characters on the screen and 27% (8/30) of the participants were neutral about knowledge of the rules to stick to when inputting data. In addition, 23% (7/30) were neutral about the form being visually appealing and with their satisfaction with the form. We calculated the quantities and the respective percentages of those who *agreed, disagreed,* and those who *did not know* or were *neutral* to the evaluation statements during the evaluation exercise (Figure 1). The figure shows that about 70% of the RAs were satisfied with the form prototypes.

**Figure 2:**
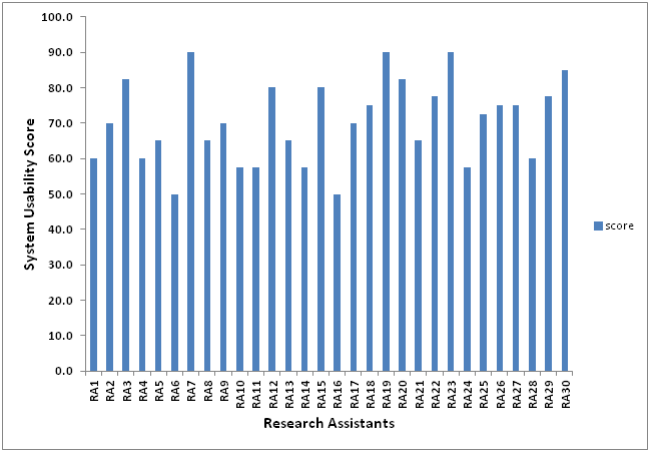
Results from the research assistants’ (RAs) evaluation using the System Usability Scale (n=30).

The individual SUSs ranged from 50 to 90 (Figure 2), with an average score of 70.4 (SD 11.7). This value was above the recommended average SUS score of 68, which showed that the RAs were fairly satisfied with the usability of the prototype. However, over 20 of the RAs felt that the form was easy to use and would like to use it more frequently, there was proper integration of various functions in the form, and they felt very confident about using the form. The same number of participants did not find the form unnecessarily complex, and neither was there any inconsistency in the form. For some of the statements, the number of participants who were *agreeing* and *disagreeing* was almost equal. For example, 12 felt they would need a technical person to use the form, whereas 16 did not, 12 felt the form was cumbersome to use, 15 felt otherwise, and 18 participants felt they needed to learn a few things first before using the form whereas 15 *disagreed* to that. Finally, 9 of the participants would opt not to use the form more frequently.

**Figure 3:**
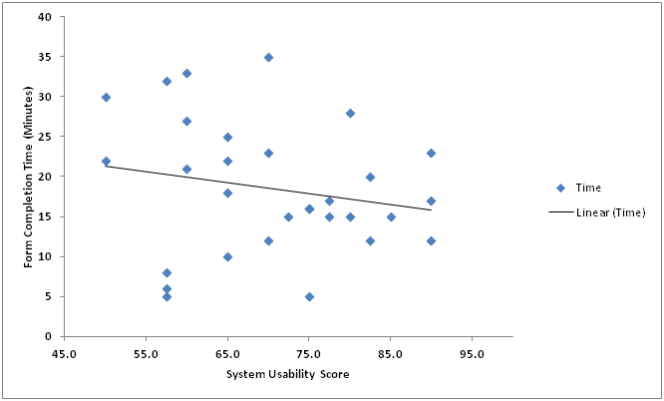
System Usability Scale compared with form completion time (minutes).

We plotted a graph to compare the association between the time it took to complete the form and the SUS scores (Figure 3). The results indicate that the time the participants took to fill the form also varied ranging from 5 to 35 min across the participants, which gave an average of 19 min overall. The direction of the relationship between the SUS score and the time is negative as shown in Figure 3. Results from the bivariate Pearson correlation we conducted indicated that the SUS score and the time taken did not have a statistically significant linear relationship because *P*=.699 which is greater than .01 for a 2-tailed test.

### Comparison of Results From the System Usability Scale and the Study Tailored Evaluation Questionnaire

Using these instruments concurrently turned out to be important because we were able to test for both usability and UX using the 2 instruments. In this study, the SUS is meant to measure usability, whereas the evaluation questionnaire is more detailed and meant to capture more of the UX after including the new design preferences.

**Figure 4:**
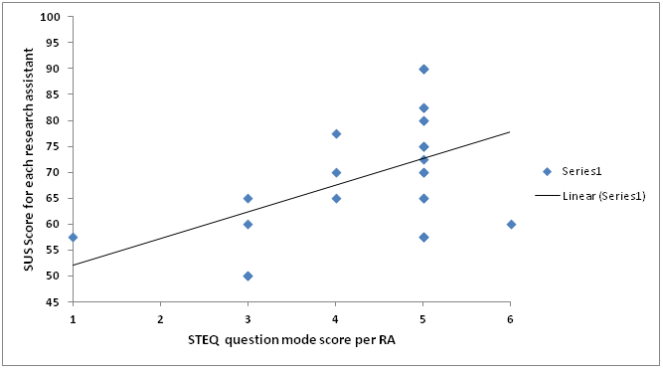
System Usability Scale (SUS) score compared with the Study Tailored Evaluation Questionnaire (STEQ) score. RA: research assistant.

Figure 4 indicates a positive relationship between the 2 variables, for example, the participants who were satisfied with the prototype (scored 4 or 5) according to the STEQ had high SUS scores and the ones who were not satisfied (scored 1 or 2) had relatively low SUS scores. The results from the bivariate Pearson correlation indicate that this relationship is significant at the .01 level for a 2-tailed test because the *P*-value is less than .01. The Pearson correlation value of .62 further signifies a strong association between the SUS score and the STEQ score.

The participants with the lowest SUS scores all found that the form was not simple to fill, easy to use, and were also not satisfied with it as depicted in the STEQ. These results could be attributed to the fact that there was a general comparison between the forms they had been using (ODK) and the high-fidelity prototype. It felt that the prototype was limiting their usage because due to missing functionality they could not freely do what they were used to doing with ODK. In general, the results from these 2 instruments are proof that the 2 evaluation methods or instruments are meant to complement each other and not to compete against each other [41].

We also note that the results for our generated affirmative STEQ do not depict any acquiescence bias because there were variations in the number of participants who *agreed* to a specific evaluation statement, meaning that not all the participants simply agreed to the evaluation statements. The percentage of participants with agreeable responses ranged from 60% (18/30), which was the lowest number, to 85% (29/30) the highest percentage (Figure 4). We also did not experience extreme response bias because the participants’ responses did not only target the extreme options on the scale but also included neutral responses as shown in evaluation statements 5, 6, 11, and 13 where the percentage of respondents were 26% (8/30), 36% (11/30), 30% (9/30), and 76% (23/30) respectively. Thus, from this questionnaire, we were still able to get what the participants felt about the data collection form.

## Discussion

### Principal Findings

Our findings from the STEQ indicated that about 70% of the responses were *agreeable* to the affirmative statements, and the alternative average SUS score was 70.4, which showed that the participants were generally satisfied with the data collection forms. The results also indicated a strong positive association between the 2 evaluation questionnaires. Using 2 evaluation methods turned out to be important because it provided an opportunity to test for both the usability of the forms and the UX. This is based on the fact that a product with good usability can generate negative UXs, hence leading to dissatisfaction, whereas a product with bad usability can generate positive experiences or satisfaction [42]. In other words, good usability will not always lead to a good UX and the reverse is true.

We used 30 participants in this study, contrary to the recommended 5 by some researchers. The justification of the number of use testers varies and is usually linked to the benefit per cost ratio [43], whereas some researchers also intimate that 5 test users are enough to detect 80% of the usability problems [44]. However, Pablo [17] suggests selecting as many users as would be representative of the target audience provided it does not affect the usability data analysis.

Usability is not an absolute concept, but is relative, dependent on the task and the user [17]. In this study, the variations in the levels of agreement with the different design features and the time taken to complete the tasks by the participants support this. The time the users spent in the evaluation process ranged from 5 to 35 min. The participants had never been involved in such an activity before, and at times found it difficult to follow the tasks while filling the form, which affected their time specifically during consultation. Some of the vocabulary particularly in the SUS may have been a bit complex to the participants, considering that usability was a new discipline to the participants.

Prototype evaluation as a means of usability testing may not necessarily identify comprehensively all the design problems in the prototype [17] because it may be hard to observe the participants diligently, attend to all their queries, and at the same time record the sessions all in one go. Thus, using prototype evaluation can be a time-consuming and error-prone task that is dependent on subjective individual variability [17]. However, errors can be managed by ensuring that there are enough observers during the exercise to support the participants where necessary, and also the tasks chosen should cater for the variability of all the participants. Using a prototype that can be accessed in an offline state would also be useful especially in areas where internet access and speeds are a problem.

### Study Limitations

Metrics from posttest evaluations do not indicate why users struggle with any design and also do not provide insight on how the design can be improved because their main focus is on tracking how users feel about using a given product [33]. Their main focus is on producing a usability score for the system rather than the identification and remediation of the specific usability issues [45]. This was true for this study as well because the RAs were not required to elaborate on why they had scored the way they did, which then leaves a gap on how best to improve the MEDCF design. There is therefore a need to identify these usability issues and remediation and give them the attention they deserve.

It is important to note that the SUS questionnaire was given after the first evaluation questionnaire, when some of the participants were probably tired and had lost their concentration, which may have had an influence on the SUS score. It was evident in some questionnaires that the users did not give much thought to what they were evaluating but ticked the same score across all the statements, for example, 1 participant who scored 50 selected *agreed* to 8 of the 10 SUS statements. This kind of evaluation certainly affects the results of the SUS score because of the alternating positive and negative statements that comprise this instrument. The SUS was deliberately designed to obtain reliable scores by alternating positive and negative statements on the same thing, that is, the UX dimension.

It was not possible to attach the users’ experience to their individual scores, because we collected the demographics data during the evaluation of the mid-fidelity prototype [29] and we did not collect it again, and yet the participants did not have unique identifiers.

The results also indicate that the participants were not satisfied with the size of the screen characters and visual appeal. One would argue that the phone had a small screen size as in some cases, one had to scroll up and down several times on the same page to fill up the content on that screen. This could have had an impact on the scores from the RAs and the subsequent results.

A reasonable amount of time was spent trying to secure an internet connection, and on getting it, the internet speed was rather slow hence affecting the prototype loading time. As a result, the participants had to work in shifts because the internet could support 5 people at a go, meaning that some of the participants had to wait for longer hours before they could finally begin the exercise. Second, Survival Pluss project has a follow-up component of their recruited mothers, and some of these RAs had prior appointments to meet these mothers at the time when we were carrying out the evaluation. This also prolonged the time taken to carry out the evaluation because some of the RAs were not available on particular days or particular times.

### Recommendations and Future Work

Tailoring OSS solutions to user-specific needs and preferences at reasonable costs is worth the effort. We thus recommend that data collectors worldwide are involved in form design and evaluation as early involvement could also help understand the potential of the group, their preferences, and the group’s appropriate design solutions.

It is also important to consider the infrastructure and the user groups in such group testing activities, for example in this case, it would be advisable to have the prototype accessible in an offline state especially in areas where internet accessibility is a challenge.

It is not always feasible for software developers to include more resource-demanding features such as rich graphics, and perhaps some elements of gamification, but it is important to note that the RAs will always have some expectations that are worth exploring and considering.

### Conclusions

Evaluating user design preferences to determine the UX using the group testing approach is not a common approach in the development of mobile data collection forms, and yet this could be one way of tailoring design to the user needs so as to cater for the diversity in context and user groups especially in rural Africa [46]. Using high-fidelity prototyping to demonstrate the design variations turned out to be a feasible and affordable form development option irrespective of the time it consumed during the evaluation process. The design features in the high-fidelity prototype that were evaluated can be a good basis when designing mobile data collection forms to improve usability and UX. In addition, adopting 2 evaluation instruments could be considered during user testing for purposes of comparing and complementing findings.

## Appendix

Multimedia Appendix 1 Screenshots showing the high-fidelity prototype.

Multimedia Appendix 2 Tasks carried out during interaction with the prototype.

## Abbreviations

DSR: design science research
MEDCF: mobile electronic data collection form
NORHED: Norwegian Programme for Capacity Development in Higher Education and Research for Development
ODK: Open Data Kit
OSS: open-source software
RA: research assistant
STEQ: Study Tailored Evaluation Questionnaire
SUS: System Usability Scale
UCD: user-centered design
UX: user experience
