# Supplementary material for: High-Fidelity Prototyping for Mobile Electronic Data Collection Forms Through Design and User Evaluation
Source: JMIR Hum Factors. 2019 Mar 22;6(1):e11852. doi: 10.2196/11852 (PMC6450481; doi:10.2196/11852)
Supplement: Multimedia Appendix 1 [file humanfactors_v6i1e11852_app1.pdf]

Multimedia Appendix 1:

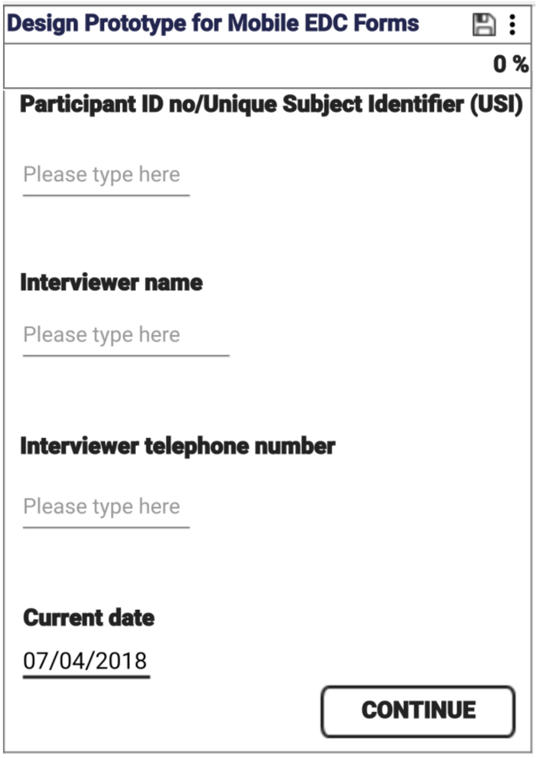

Figure 1a: The demographic section

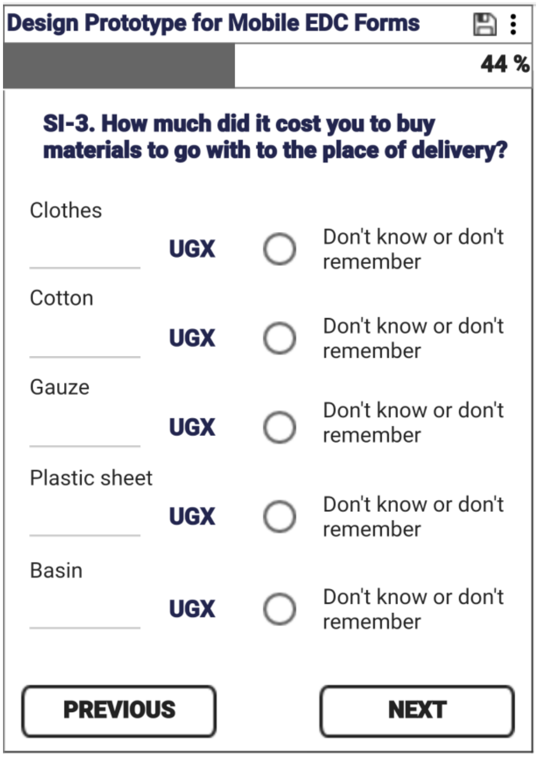

Figure 1b: The list pickers

In the demographic section (Figure 1a), the RAs were required to fill in a participant ID, interviewer name and the interviewer telephone number, the date automatically updated. The participant ID had to be between 1000-9999, the interviewer name included the first and the last name and had to consist of at least 10 letters, and the telephone number was comprised of 10 numbers. It was not possible for the user to proceed without filling in these fields correctly. The progress status on continuation to the next screen was represented by an increase in percentage, and the users could navigate the prototype by either swiping or by using the navigation buttons in the form.

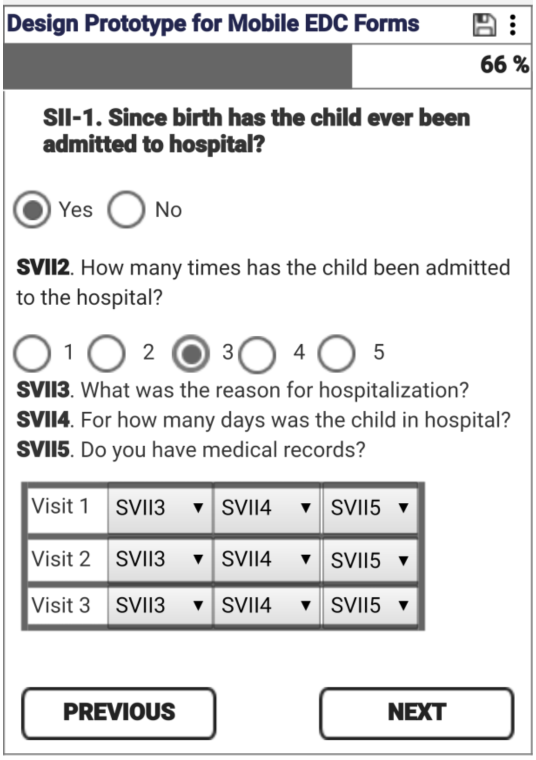

Figure 2a: The child's sickness record

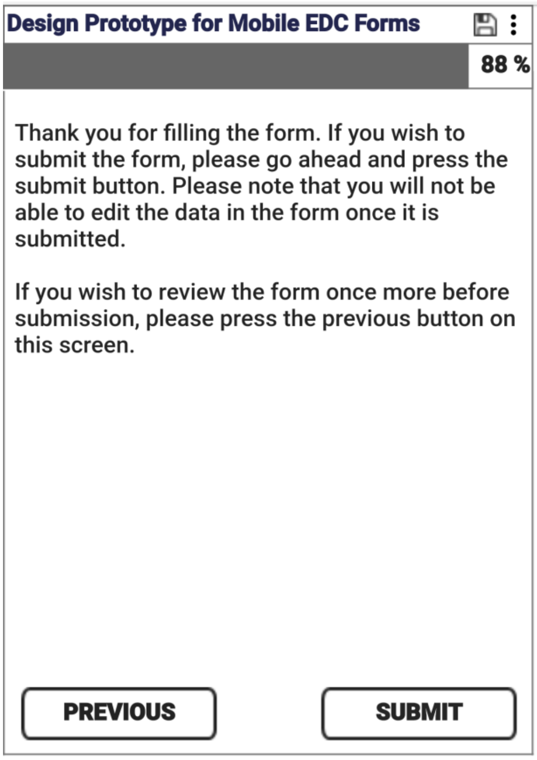

Figure 2b: The submission screen

Section I of the prototype was concerned with list pickers (single choice and multiple choice pickers) (Figure 1b). In the single choice list picker, the participants were required to select only one option here, and if they chose the 'other' option, they were required to specify that option. The participants could pick more than one option in the multiple choice option, and could still specify the 'other option' if selected. It was not possible to continue to the next screen unless the fields were filled appropriately. Still in this section, members were required to fill in the cost of the materials they used when going to hospital. In case they didn't know or couldn't remember how much they spent, the 'don't know' or 'don't remember' option was available. Since this was being tested in Uganda, the users were required to type a value of 4 digits or more. It was also not possible to select 'don't know' and to type a cost against the same item. Every item in the form either had to have a cost or a 'don't know' or 'don't remember' against it before the user could continue to the next screen.

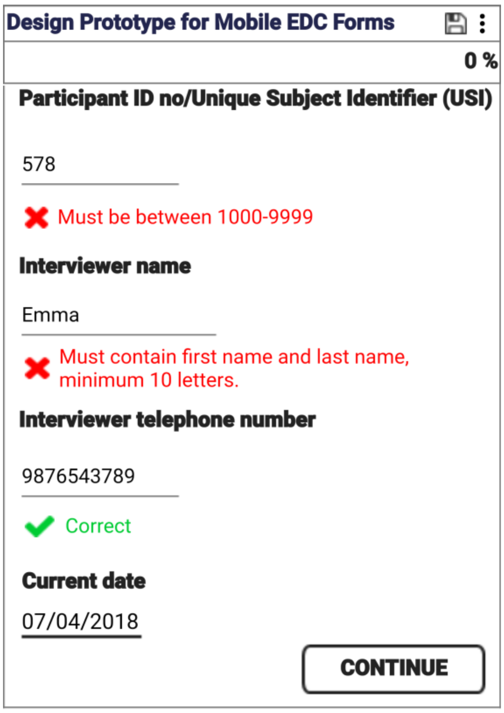

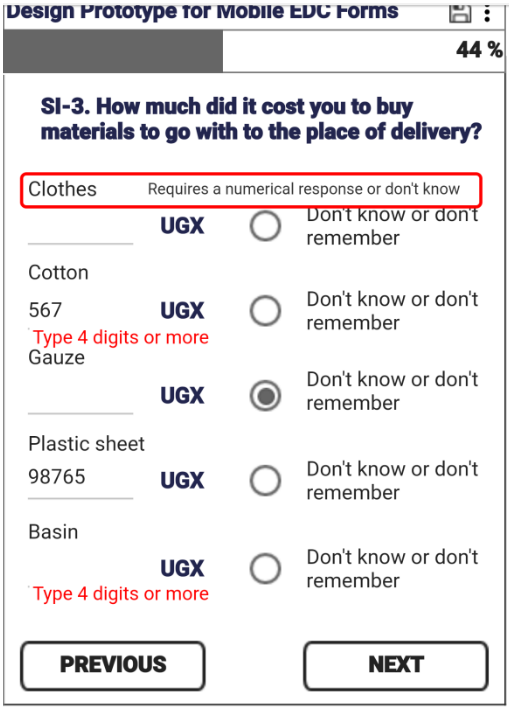

Figure 3a: Validation testing screen Figure 3b: Screen indicating feedback

Section II of the prototype showed different table designs capturing a child's sickness record (Figure 2a). The participant was required to indicate if a child had been hospitalized or not. If the child had not been hospitalized before, the participant would be requested to submit the form. However, if the child had been admitted, the number of times a baby had been hospitalized, the reason for the hospitalization, the number of days for each hospitalization and whether the medical records were available or not were recorded. Each of these options had a drop down list from where the RAs were required to select what they felt the correct option was.

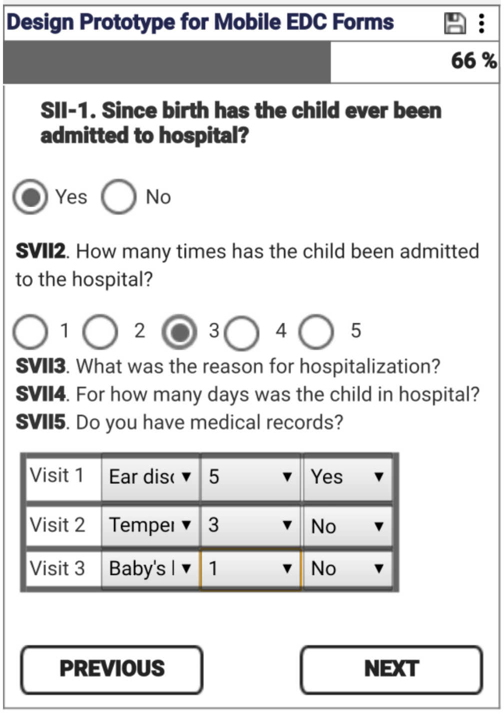

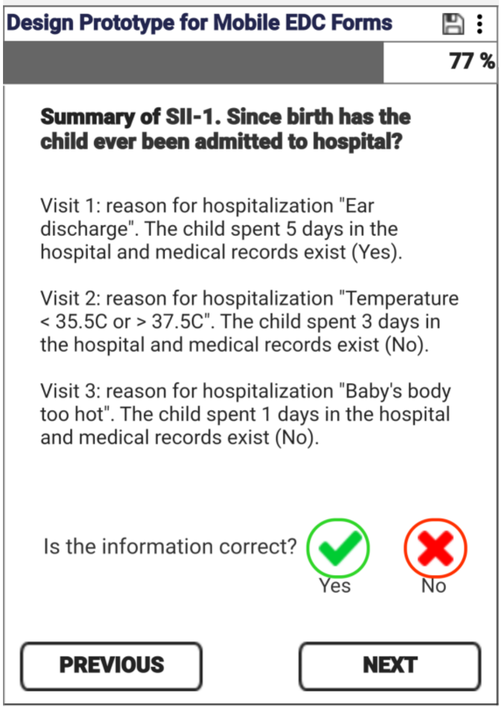

Figure 4a: Screen showing logic implementation Figure 4b: Summary of information from screen 4a
