# Supplementary material for: High-Fidelity Prototyping for Mobile Electronic Data Collection Forms Through Design and User Evaluation
Source: JMIR Hum Factors. 2019 Mar 22;6(1):e11852. doi: 10.2196/11852 (PMC6450481; doi:10.2196/11852)
Supplement: Multimedia Appendix 2 [file humanfactors_v6i1e11852_app2.pdf]

Multimedia Appendix 2

Table 1: Tasks for the high fidelity prototype evaluation of MEDCFs

Task	Task description
1.	• Enter a participant ID no with 3 digits.<br>• Type your first name<br>• Fill in your telephone number
2.	• Press the next button without selecting any option<br>• Select 'Other specify'<br>• Press 'next' without typing anything for 'other specify'<br>• Press the next button without selecting any option<br>• Select as many options as you want<br>• Select 'Other' and proceed
3.	• Type any text under clothes, cotton and gauze.<br>• Select 'don't know' for plastic sheet and basin and press next<br>• Fill out the amount spent on clothes, cotton and gauze.<br>• Select 'don't know' on plastic sheet, and leave basin option empty.<br>• Press next
4.	• Select 'No' for child admission<br>• Press the 'previous' button twice and go back to question SII-1<br>• Select 'Yes' for child admission<br>• Select '3' for number of admissions<br>• Fill the rest of the table appropriately
5.	• Check the summary generated on the next screen.<br>• Proceed

The first task the users carried out was to evaluate the validation process of the data being entered, and this was based on the validation time and the way the validation feedback was presented. The users had to enter a participant ID with 3 digits, to type their first name and their telephone number (Figure 3).

The second task tested the use of the 'other' option in the list picker options (Figure 3). For the single choice list picker, the users had to press the 'next' button without selecting any option, select 'other specify', and to press the 'next' button without typing anything for 'other specify'. For the multiple choice list pickers, the users were required to press the 'next' button without selecting any option, to select as many options and the 'other' option and then proceed.

The third task tested error handling which involved error messaging (Figure 3a) and recovery from error (Figure 3b). The users had to type text instead of numbers under each of the clothes, cotton and gauze items, select 'don't know' for plastic sheet and basin and press the 'next' button. They were then required to type the amount of money spent on clothes, cotton and gauze, then select 'don't know' for plastic sheet, leave basin empty and select the 'next' button.

The fourth task tested the logic implementation in the form (Figures 4a and 4b). The users were required first to select 'no' for child admission, and press the previous button to go back to the start of the question. They were then required to select 'yes' for child admission, select '3' for number of admissions and fill the rest of the form appropriately. They then checked the summary generated from the data entered on the child sickness and proceeded to submit the form.
